# Population Pharmacokinetics of High-Dose Tigecycline in Patients with Sepsis or Septic Shock

**DOI:** 10.1128/AAC.02273-17

**Published:** 2018-03-27

**Authors:** Agnieszka Borsuk-De Moor, Elżbieta Rypulak, Beata Potręć, Paweł Piwowarczyk, Michał Borys, Justyna Sysiak, Dariusz Onichimowski, Grzegorz Raszewski, Mirosław Czuczwar, Paweł Wiczling

**Affiliations:** a Department of Biopharmaceutics and Pharmacodynamics, Medical University of Gdańsk, Gdańsk, Poland; b 2nd Department of Anaesthesiology and Intensive Therapy, Medical University of Lublin, Lublin, Poland; c Department of Anesthesiology and Intensive Care, Faculty of Medical Sciences, University of Warmia and Mazury, Olsztyn, Poland; d Department of Physiopathology, Institute of Rural Health, Lublin, Poland

**Keywords:** pharmacokinetics, population pharmacokinetics

## Abstract

Tigecycline is a glycylcycline often used in critically ill patients as the antibiotic of last resort. The pharmacokinetics (PK) of tigecycline in intensive care unit (ICU) patients can be affected by severe pathophysiological changes so that standard dosing might not be adequate. The aim of this study was to describe population PK of high-dose tigecycline in patients with sepsis or septic shock and evaluate the relationship between individual PK parameters and patient covariates. The study population consisted of 37 adult ICU patients receiving a 200-mg loading dose of tigecycline followed by multiple doses of 100 mg every 12 h. Blood samples were collected at 0.5, 2, 4, 8, and 12 h after dose administration. A two-compartment model with interindividual (IIV) and interoccasion (IOV) variability in PK parameters was used to describe the concentration-time course of tigecycline. The estimated values of mean population PK parameters were 22.1 liters/h and 69.4 liters/h for elimination and intercompartmental clearance, respectively, and 162 liters and 87.9 liters for volume of the central and peripheral compartment, respectively. The IIV and IOV in clearance were less than 20%. The estimated values of distribution volumes were different from previously published values, which might be due to pathophysiological changes in ICU patients. No systematic relationship between individual PK parameters and patient covariates was found. The developed model does not show evidence that individual tigecycline dosing adjustment based on patient covariates is necessary to obtain the same target concentration in patients with sepsis or septic shock. Dosing adjustments should be based on the pathogens, their susceptibility, and PK targets.

## INTRODUCTION

Tigecycline is a glycylcycline antibiotic approved by the FDA for the treatment of complicated skin and skin structure infections (cSSSI), complicated intraabdominal infections (cIAI), and community-acquired bacterial pneumonia (CABP) (1). The pharmacokinetics (PK) of tigecycline is characterized by a large volume of distribution at steady state (7 to 10 liters/kg of body weight) compared to other antimicrobials and dose-independent clearance (CL) (2). The drug is eliminated mainly by fecal excretion of unchanged tigecycline with a minor renal elimination of unchanged drug, glucuronide conjugates, and *N*-acetyl-9-aminominocycline metabolite (3). It is highly bound to plasma proteins and exhibits atypical nonlinear protein binding (4).

The FDA issued a boxed warning for increased risk of death with tigecycline treatment for FDA-approved and nonapproved uses, but the cause of higher mortality was not established (5). Consequently, the FDA advises the use of tigecycline only in situations where alternative treatments are not suitable (5). Due to a shortage of other effective antimicrobials and the wide spectrum of tigecycline *in vitro* activity, including multidrug-resistant (MDR) and extensively drug-resistant (XDR) pathogens, tigecycline is often used off label in critically ill patients as the antibiotic of last resort (1, 6–8). As sepsis and septic shock are associated with high morbidity and mortality, optimization of antibiotic therapy based on informative population models might play a role in increasing a patient's chances of survival (9).

Standard dosing of antimicrobials results in target drug concentrations in mild to moderately ill patients, but in critically ill patients the pathophysiological changes may influence drug PK and consequently affect required dosing (10, 11). Changes in PK of patients with sepsis or septic shock include changes in clearance caused by increased cardiac output or organ failure and shifts in volume of distribution as a result of increased vascular permeability or altered protein binding (12). Changes in physiology that alter the PK can also be caused by medical interventions such as mechanical ventilation, continuous renal replacement therapy (CRRT), extracorporeal membrane oxygenation (ECMO), etc. (10, 13). As the state of the patient changes over time, dosing should be adjusted accordingly. To do so, one has to identify the relationship between measurable patient covariates and pharmacokinetic parameters.

The recommended dosage regimen for tigecycline is a 100-mg initial dose, followed by 50 mg every 12 h (1). However, dosing recommended in package inserts might be insufficient in critically ill patients (14) and result in underdosing of tigecycline (4, 15). Adequate dosing of antibiotics in patients with sepsis or septic shock is of special importance, as underdosing can lead to insufficient antimicrobial activity and negatively affect the patient's outcomes (10, 14). Ramirez et al. (16) investigated the use of higher doses of tigecycline (150 mg followed by 75 mg every 12 h and 200 mg followed by 100 mg every 12 h) and proved them to be more effective than imipenem/cilastatin treatment for hospital-acquired pneumonia without adverse effects in groups with high doses of tigecycline. Similar findings were published by De Pascale et al. (17), who retrospectively compared standard tigecycline dosing with a 200-mg loading dose followed by 100 mg every 12 h and found improved outcomes for patients with MDR Gram-negative ventilator-associated pneumonia in the higher tigecycline dosing group.

The objectives of this study were to describe population pharmacokinetics of high-dose tigecycline in patients with sepsis or septic shock treated in two tertiary medical/surgical intensive care units (ICUs) and examine the relationship between patient characteristics and individual PK parameters in order to propose dose adjustments according to patient covariate values.

## RESULTS

The analyzed data consisted of 942 observations of tigecycline concentrations obtained from 37 patients. Two measurements were identified as outliers during the model-building process (conditional weighted residuals [CWRES], >5) and were excluded from the analysis. A summary of the patients characteristics is presented in Table 1. The changes in time-dependent covariates in the population during 3 subsequent days of therapy are shown in Fig. S1 in the supplemental material.

**TABLE 1:** Demographic and medical condition characteristics of the patients in the study population^*a*^

Parameter (unit)	Median (range) (*n* = 37)
Age (yr)	61 (25–79)
Weight (kg)	80 (50–129)
Height (cm)	175 (158–190)
Male/female (no.)	26/11
Death/survival (no.)	23/14
ECMO, no/yes (no.)	35/2
CRRT, no/yes/started during therapy (no.)	6/30/1
Dialysis (ml/kg)	23.8 (14.2–40.0)
Ultrafiltration (ml/kg/h)	1.54 (0.34–6.6)
ELWI (ml/kg)	9 (5–41)
Cardiac output (liters)	7.49 (2.55–15.8)
SOFA score	13 (2.0–21)
Procalcitonin concn (μmol/liter)	8.22 (0.16–122)
Albumin concn (g/dl)	2.2 (1.5–3.6)

a Values are expressed as medians and ranges for continuous variables and as counts for categorical variables.

**FIG 1:**
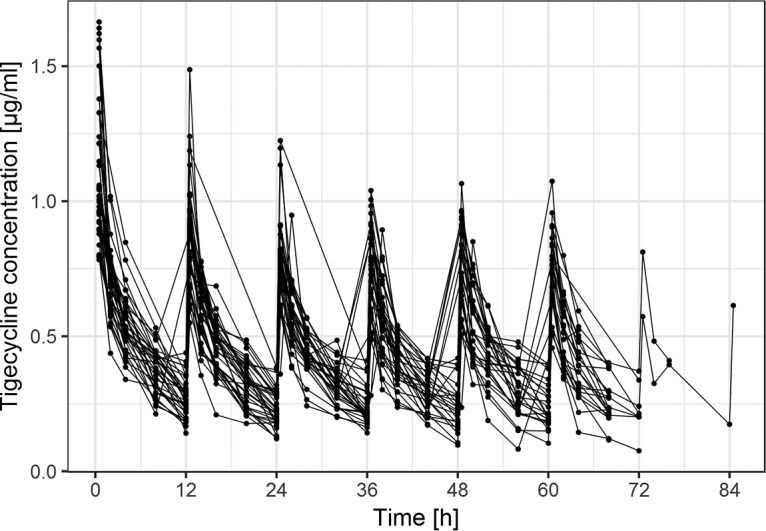
Individual tigecycline concentration-time profiles of the studied population.

The raw concentration data are shown in Fig. 1. A two-compartment disposition model was used to describe the available data. Interindividual variability was estimated for CL, volume of distribution of the central compartment (*V*_1_), and volume of distribution of the peripheral compartment (*V*_2_), but it was not possible to estimate the interindividual variability (IIV) for the intercompartmental clearance (*Q*_2_). A visual covariate search was performed for this model, but no systematic relationship was found.

As data were available for each patient on multiple dosing occasions, interoccasion variability (IOV) in individual PK parameters was investigated. In the final population model, IOV was estimated for individual CL and *V*_2_ parameter values.

Table 2 shows parameter estimates of the final population PK model of tigecycline along with their bootstrap estimates. All PK parameters and intersubject, interoccasion, and residual error variances were estimated with a relative standard error (RSE) of less than 66%. The estimates of the model parameters fell very close to the median estimates obtained from bootstrap analysis, which proves the final model estimates are unbiased.

**TABLE 2:** Final model parameter estimates

Parameter^*a*^	Estimate	RSE (%)	Shrinkage (%)	Bootstrap analysis	
				Median	90% CI^*b*^
Mean population parameters (θ)					
θ_CL_ (liters/h)	22.1	3.16		22.1	20.9–23.2
θ_V1_ (liters)	162	5.3		163	150–176
θ_Q_ (liters/h)	69.4	32.6		67.3	41.9–98.4
θ_V2_ (liters)	87.9	8.67		87.6	76.1–101
Interindividual variability (ω^2^)					
ω^2^_CL_ (% CV)	17.3	19	7.3	17.1	14.2–19.7
ω^2^_V1_ (% CV)	19.2	29.2	6.7	19.1	14.3–23.7
ω^2^_Q_ (% CV)	0 FIX^*c*^				
ω^2^_V2_ (% CV)	38.7	40.8	22.4	37.4	20.4–48.5
Interoccasion variability (π^2^)					
π^2^_CL_ (% CV)	14.4	35		14.2	9.4–18.1
Occasion 1			27.4		
Occasion 2			38.5		
Occasion 3			52.4		
Occasion 4			42.6		
Occasion 5			35.4		
Occasion 6			48.4		
Occasion 7			94.9		
Occasion 8			99.8		
π^2^_V2_ (% CV)	20.8	66.4		21.7	0.200–30.9
Occasion 1			40.2		
Occasion 2			50.2		
Occasion 3			58.5		
Occasion 4			56.4		
Occasion 5			52.9		
Occasion 6			57.2		
Occasion 7			90.6		
Occasion 8			99.6		
Residual error model					
σ_add_ (μg/ml)	0.0210	0.41		0.0224	0.000209–0.0357
σ^2^_prop_ (% CV)	13.0	17.7		12.7	8.89–16.1

a σ_add_, additive residual random error; σ^2^_prop_, variance of proportional residual random error.

b 90% CI (confidence intervals) of the parameter estimates were derived from a nonparametric bootstrap analysis (*n* = 1,000; unsuccessful, 1).

c 0 FIX indicates that parameter value was fixed at 0 and not estimated.

**FIG 2:**
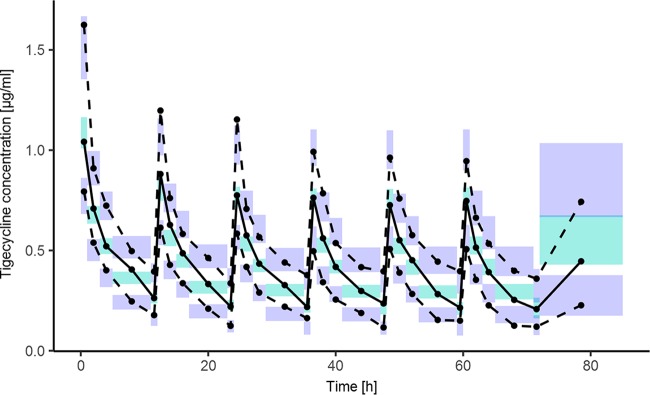
Visual predictive check showing the simulation-based 90% confidence intervals around the 5th, 50th, and 95th percentiles of the PK data in the form of turquoise (50th) and violet (5th and 95th) areas; the corresponding percentiles from the observed data are plotted in black color.

Goodness-of-fit plots of the final model are presented in Fig. S2. The individual and population predictions versus observed concentrations are relatively symmetrically distributed around the line of identity. The conditional weighted residuals versus time and versus individual predicted concentrations do not show any trend and are relatively evenly distributed around zero. The visual predictive check (VPC) plot presented in Fig. 2 indicates that both the central tendency of the data and the variability at a particular sampling time were recaptured very well. The individual predicted concentration-versus-time profiles were very close to the experimental data, as presented in Fig. S3.

In the final model, the typical values of elimination and intercompartmental clearance were 22.1 liters/h and 69.4 liters/h. The typical values of volume of central and peripheral compartment were 162 liters and 87.9 liters. The interindividual variability was intermediate for CL (17.3%) and *V*_1_ (19.2%) and higher for *V*_2_ (38.7%). The interoccasion variability for CL and *V*_2_ was 14.4% and 20.8%. The shrinkage for η (variable used to model differences between the individuals) was low (7.3% for CL, 6.7% for *V*_1_, and 22% for *V*_2_), while for κ (variable used to model differences between occasions within individuals) it was generally higher, reaching >90% for occasions 7 and 8, where only a few observations were available.

**FIG 3:**
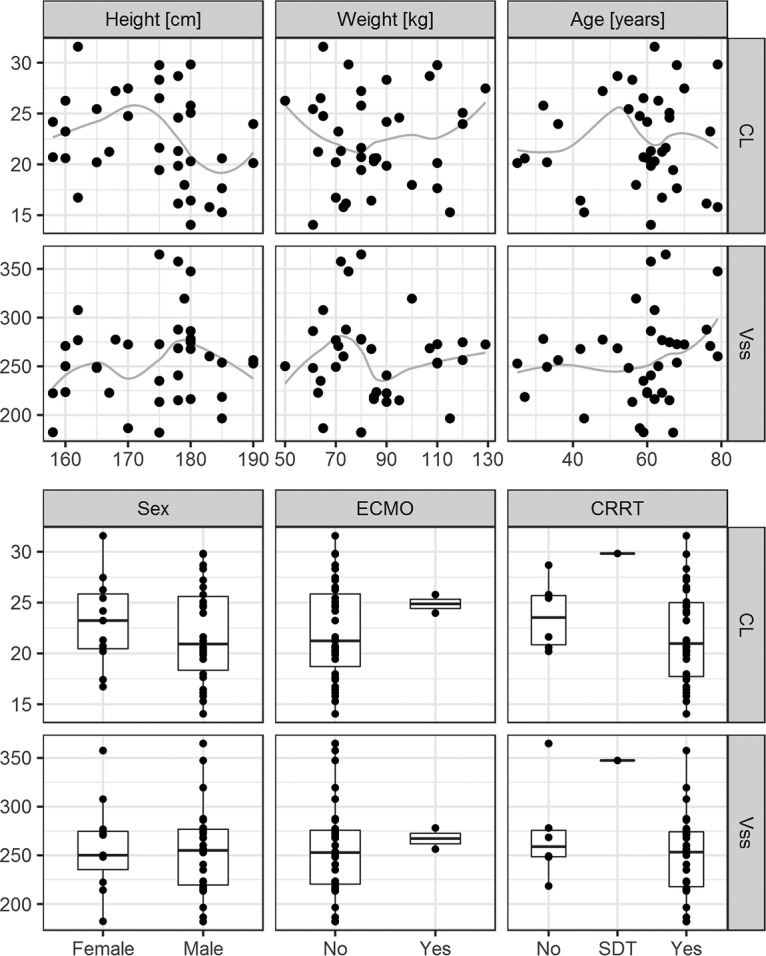
Estimates of individual PK parameters from the final model in relation to time-independent continuous covariates and categorical covariates. The gray lines indicate the trend in the data (using locally weighted scatterplot smoothing [LOESS]). CL, clearance; *V*_ss_, volume of distribution at steady state; ECMO, extracorporeal membrane oxygenation; CRRT, continuous renal replacement therapy; SDT, started during therapy.

**FIG 4:**
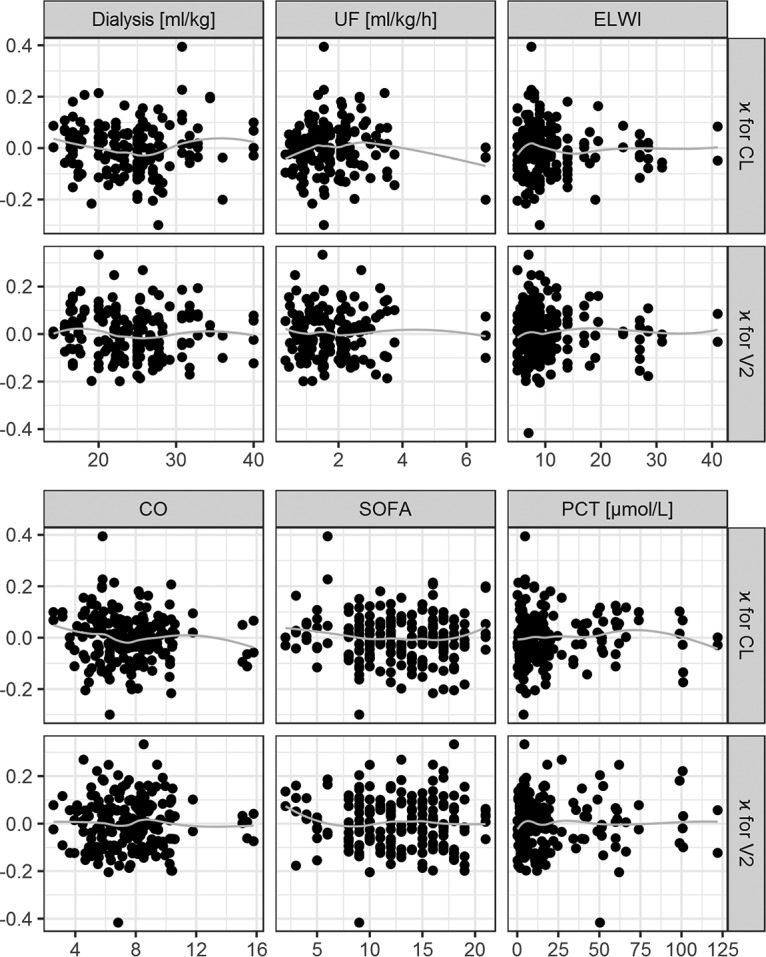
Individual estimates for kappa of the final PK parameters in relation to time-dependent covariates. The gray lines indicate the trend in the data (using LOESS). κ, deviation of individual parameters between occasions; CL, clearance; *V*_2_, volume of distribution of the peripheral compartment; ELWI, extravascular lung water index; CO, cardiac output; SOFA, sequential organ failure assessment score; PCT, procalcitonin concentration.

The relationships between the individual values of CL and volume of distribution at steady state (*V*_ss_) and time-independent covariates are presented in Fig. 3. The relationships between the estimates of κ for the individual CL and *V*_2_ values and the time-dependent covariates are presented in Fig. 4. The relationships between the estimates of η for CL, *V*_1_, and *V*_2_ versus individual values of the time-independent covariates and median values across occasions of time-dependent covariates are presented in Fig. S4 to S6. The lack of any regular trend in the data indicates that the analyzed covariates cannot explain the remaining unexplained between-patient and between-occasion variability. The relationship between the individual values of CL and variables calculated based on weight and height, i.e., body surface area and body mass index (BMI), was additionally explored, but no trend was discovered.

## DISCUSSION

**FIG 5:**
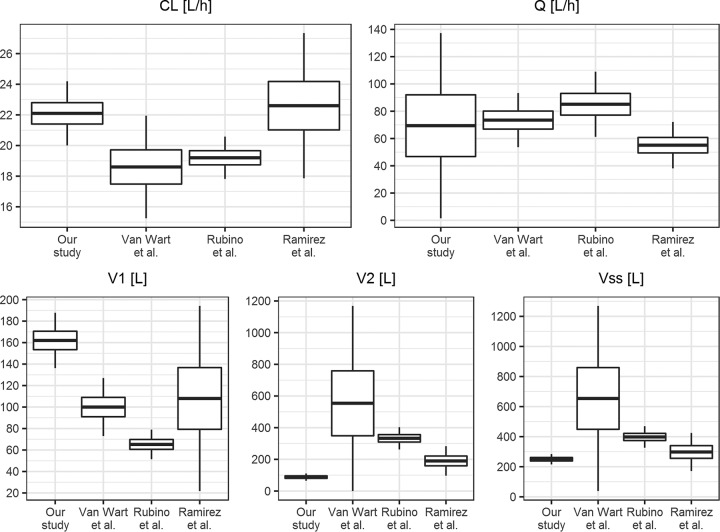
Comparison of the population PK parameter values estimated in our study and the studies of Van Wart et al. (18), Rubino et al. (19), and Ramirez et al. (16). The boxplots show the mean values and standard deviations of the parameter. For the study by Rubino et al. (19), the standard deviations of the mean clearance estimates were obtained from bootstrap results. CL, clearance; *V*_ss_, volume of distribution at steady state; *Q*, intercompartmental clearance; *V*_1_, volume of distribution of the central compartment; *V*_2_, volume of distribution of the peripheral compartment.

The comparison of the mean PK parameter values obtained in this study with selected literature values (16, 18, 19) is presented in Fig. 5.

The estimated mean value of clearance is consistent with published studies (16, 18–20), and it is also in good agreement with tigecycline product information (CL of 24 liters/h) (1). A study by Xie et al. (21) of critically ill patients reports lower clearance of 7.5 liters/h; however, this value was estimated based on small population (10 patients). Overall, the expected area under the concentration-time curve and average concentration achieved after tigecycline administration is consistent across the studies. Good estimation of clearance is the most important for safety and efficacy of therapy, especially in the context of the boxed warning issued for tigecycline by the FDA (5). In our study, the clearance IIV (17.3%) was estimated to be approximately two times smaller than those in other studies (16, 18, 19), but part of the variability in clearance (14.4%) was assigned to IOV. This suggests that the variability in tigecycline clearance is caused by differences between patients as well as differences within a patient on various occasions. Small interindividual and interoccasion variabilities in clearance suggest that to obtain the same target concentration in all patients, uniform dosing of tigecycline is sufficient in critically ill patients. To find a clinically significant relationship between covariate and parameter, the relationship should lead to reduction in the variability of this parameter by 20%. In our case, with IIV and IOV lower than 20%, it is not possible.

The values of intercompartmental clearance are similar between the studies, as shown in Fig. 5, but were estimated with different precision. The lower precision of *Q* value estimates in our study is due to having little information about this parameter from the applied sampling design.

**FIG 6:**
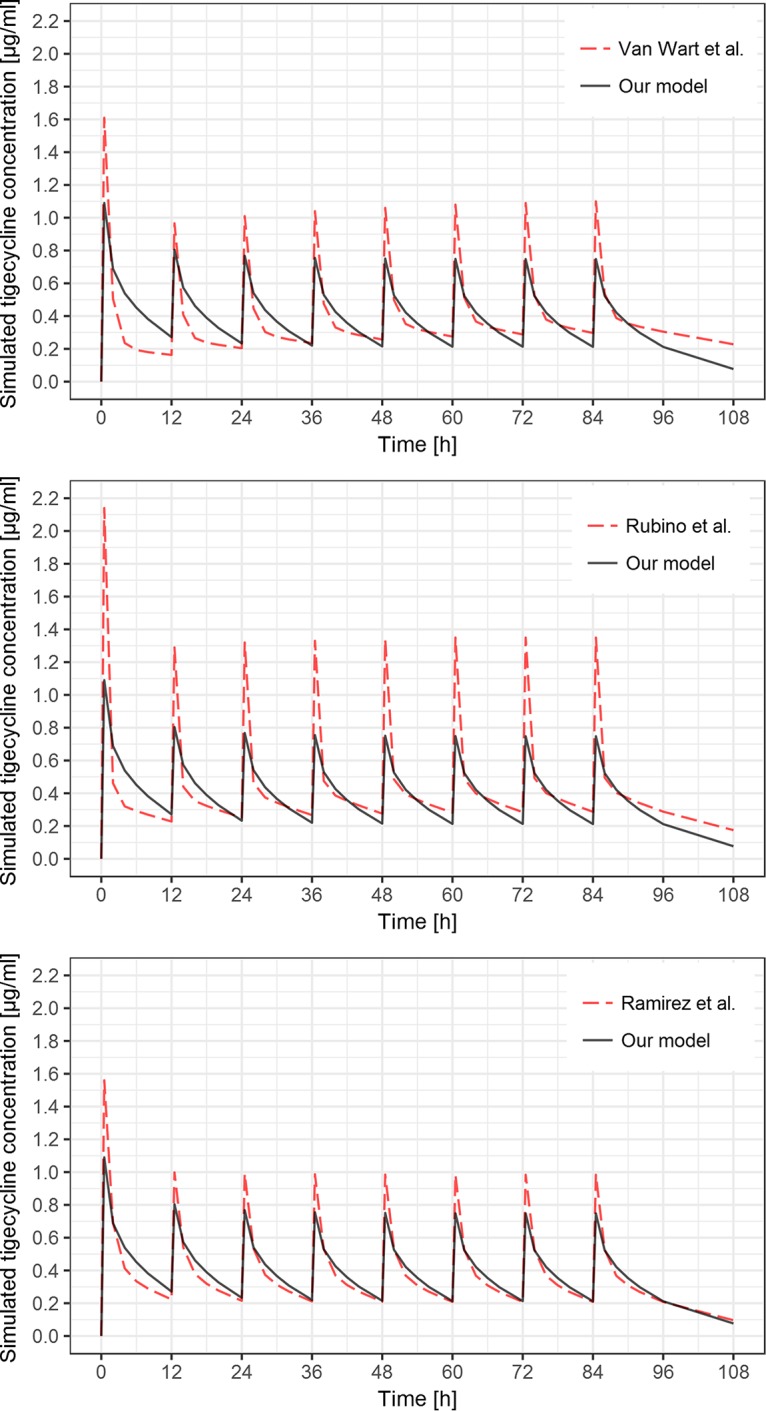
Simulation of tigecycline concentrations after 200-mg loading dose and seven subsequent 100-mg doses in 12-h intervals based on typical population PK parameters from the model developed in this study (black solid lines) and the models published by Van Wart et al. (18), Rubino et al. (19), and Ramirez et al. (16) (red dashed lines).

The values of volumes of distribution differ between studies. Mean *V*_ss_ in our study is 250 liters, which is comparable to the value of 298 liters estimated by Ramirez et al. (16) but very different from the studies by Rubino et al. (19) and Van Wart et al. (18), which can be observed in Fig. 5. Additionally, the study on healthy subjects reports *V*_ss_ between 490 and 700 liters in a 70-kg patient (20). Precise estimates of the volume of the peripheral compartment require very long sampling schedules, since they are based on the terminal part of the concentration-time profile. In our study, the sampling after the last dose is rather short, which might cause imprecise and biased *V*_2_ estimates. Even though the *V*_2_ value in the study by Van Wart et al. (18) is very high, the precision of this estimate is poor. The differences in the values of distribution volumes of central and peripheral compartments can be observed in Fig. 6, where simulation of tigecycline concentrations based on parameters from 3 studies with dosing applied in our study is presented. The value of *V*_1_ in our study is approximately 1.5-fold higher than that in the study by Van Wart et al. (18) and 2.5-fold higher than that in the study by Rubino et al. (19). This determines lower predicted peak concentrations after dose administration in our study and lower peak-trough fluctuation after multiple administrations. The value of the volume of the peripheral compartment in our study is severalfold lower than the literature values (18, 19), which results in lower drug accumulation after multiple administration (accumulation ratio of 1.57 in our study compared to 4.00 [18] and 2.55 [19]). The shift between the distribution volumes in our study might occur due to changes in physiology caused by the sepsis/septic shock of the patients in the analyzed population. Increased capillary permeability in sepsis causes the shift of the fluids from blood vessels to interstitial space, which can increase the volume of distribution of the central compartment (9). The hypoalbuminemia present in critically ill patients can also affect the volume of distribution, especially since tigecycline is highly bound to proteins and shows nonlinear plasma protein binding (4). This is not supported by the study of critically ill patients by Xie et al. (21), where the estimated value of *V*_1_ was approximately half of the value estimated in our study (72.5 liters and 162 liters, respectively). Tigecycline is a lipophilic antibiotic, and as such its volume of distribution at steady state should not be altered due to physiological changes of the patients in the ICU (12).

None of the covariates showed a clear relationship with the individual values of PK parameters, which is consistent with the results of Ramirez et al. (16) but not with those of two other studies, which included the relationship of CL versus body surface area and creatinine clearance (19) and CL versus weight, creatinine clearance, and gender (18) in the final model. In the study by Xie et al. (21), BMI was included in the model as a linear predictor of CL, but this inclusion was not supported by improvement in log likelihood value. On the other hand, no relationship between CL and body weight was reported in a study of obese patients by Pai (22). Honore et al. (23) suggested the influence of CRRT on tigecycline PK; however, the group of patients who did not receive CRRT in this study was too small to assess the impact of CRRT on individual PK parameters.

Individualized dosing of antimicrobials based on patient characteristics is important for the safety and efficacy of therapy, but the main issue for the clinician is to determine and obtain PK targets for the pathogen, which are based on the MIC (14). Since our study suggests that there is no strong relationship between tested patient covariates and individual PK parameters for tigecycline in critically ill patients, dosing adjustments should be focused on identification of pathogens, their susceptibility, and determination of PK target. With identified target plasma concentration, the dose of tigecycline can be calculated based on estimated PK parameters from our model.

### Conclusions.

The population PK model was successfully developed to describe the time course of tigecycline concentrations in patients with sepsis or septic shock. None of the available patient's covariates was identified to explain part of the IIV or IOV in the pharmacokinetic parameters, therefore no individual dosing adjustment could be proposed based on the available patient covariates. Low interindividual and interoccasion variabilities in clearance suggest that, assuming the same target concentration in all patients, uniform dosing in this population is sufficient and dosing adjustments should be based on the pathogens, their susceptibility, and PK targets. The model can be useful for further analysis of tigecycline exposure-response relationships in critically ill patients.

## MATERIALS AND METHODS

### Patients and study design.

This was a prospective, observational cohort study investigating the pharmacokinetics of tigecycline in adult patients admitted to two tertiary medical/surgical ICUs in Lublin and Olsztyn, Poland. Ethical approval was obtained from the Bioethics Committee of the Medical University of Lublin. The inclusion criteria for the study were the following: age of 18 to 75 years, sepsis or septic shock of both medical and surgical origin at admission to the ICUs, and suspected nosocomial infection with MDR or XDR strains, requiring the implementation of empirical broad-spectrum antibiotics according to the local antimicrobial prescribing policy. Patients were excluded from the study if they had no life-threatening condition at the time of onset of symptoms of infection, were diagnosed with HIV infection or terminal cancer, displayed intolerance or allergy to tigecycline in the past, had high probability of bacterial infection with tigecycline-resistant strains (e.g., Pseudomonas aeruginosa), and received tigecycline up to 3 months before being screened. Each patient received an initial dose of 200 mg of tigecycline in a short 30-min infusion, followed by multiple doses of 100 mg in 30-min infusions every 12 h. The patients included in the study received 2 to 8 doses of tigecycline for 1 to 4 consecutive days. Arterial blood samples (2 ml) for PK analysis were collected into heparinized test tubes at 0.5, 2, 4, 8, and 12 h after each tigecycline administration. Red blood cells were precipitated and removed by centrifugation at 10,000 rpm for 10 min. Blood plasma was collected and frozen at −80°C until used.

### Assay.

The analytical measurements of plasma samples were performed using a Dionex chromatographic system (Sunnyvale, CA) equipped with a UVD340S diode array UV detector and gradient pump P580 LPG LC-6A. The samples were injected using a Rheodyne 7725 loop injector with an effective volume of 20 μl. The detailed chromatographic parameters of the analytical method are described in the supplemental material.

The analytical method was validated in terms of linearity, limit of detection (LOD) and quantification (LOQ), precision, and accuracy. The method was linear over a concentration range from 0.078 to 2.5 μg/ml. The LOD and LOQ were below 0.02 μg/ml and below 0.078 μg/ml, respectively. Over the range of concentrations from 0.078 to 2.5 μg/ml of tigecycline, the intra- and interday accuracies ranged from 98.4 to 103.1, and coefficients of variation (CVs) were between 0.6 and 3.8%. The detailed procedure of the method validation is described in the supplemental material.

To determine tigecycline's concentrations, plasma samples were prepared for analysis with the following method: 200 μl of plasma was pipetted into 2.5-ml plastic centrifugal filter devices (0.22-μm GV Durapore centrifugal filter; Millipore Corporation, Billerica, MA, USA), to which 200 μl of 0.023 M phosphate buffer solution and 400 μl of acetonitrile were added and vortex mixed for 1 min. After centrifugation (at 10,000 × *g* for 10 min), the organic layer was removed and 20 μl of the aqueous phase was injected into the high-performance liquid chromatography (HPLC) system.

### Pharmacokinetic modeling.

Population nonlinear mixed-effects modeling was conducted using NONMEM software (version 7.3; Icon Development Solutions, Ellicott City, MD, USA), GNU Fortran 95 compiler (GCC 4.6.0), and Wings for NONMEM (WFN741; http://wfn.sourceforge.net). The first-order conditional estimation method using the ADVAN 3 TRANS 4 routine with η-ε interaction was employed throughout the model-building procedure. The R computing environment (R Core Team 2015) was used for data processing and visualization.

The minimum value of the NONMEM objective function (OFV), typical goodness-of-fit diagnostic plots, and evaluation of the precision of pharmacokinetic parameter and variability estimates were used to discriminate between various models during the model-building process. For two nested models, the difference in OFV is equal to minus twice the log likelihood and approximately χ^2^ distributed. A difference in OFV of 3.84 corresponds to a significance level (*P* value) of <0.05 for one additional parameter. Goodness-of-fit plots included plots of the observed concentrations versus population and individual predicted concentrations and plots of the conditional weighted residuals (CWRES) versus individual predicted concentrations and time. A nonparametric bootstrap analysis was performed to evaluate the uncertainty of final model parameters. The model performance was assessed by means of visual predictive check (VPC). Based on literature (16, 18, 19) and visual data inspection, a two-compartment model was used to describe plasma tigecycline concentration-time profiles. The model was parametrized in terms of clearances (CL and *Q*, denoting metabolic intercompartmental clearances) and volumes of distribution (*V*_1_ and *V*_2_, denoting the volumes of distribution of the central and peripheral compartment, respectively). The sum of *V*_1_ and *V*_2_ is the volume of distribution at steady state (*V*_ss_).

Interindividual variability (IIV) and interoccasion variability (IOV) of the PK parameters were modeled in terms of η and κ variables, respectively. The η variables were used to model differences between the individuals, and κ variables were used to model differences between occasions within individuals. The η and κ variables were assumed to have log-normal distributions with a mean of 0 and variances ω^2^ and π^2^, respectively. The IIV and IOV variances were assumed to be constant across occasions. The individual value of a PK parameter on a certain occasion was defined as *P_i_*_,*k*_ = θ*_P_* exp(η*_P_*_,*i*_) exp(κ*_P_*_,*i*,*k*_), where *P_i_*_,*k*_ is the individual PK parameter on a certain occasion, *θ_P_* is the typical value of this PK parameter in the population, η*_P_*_,*i*_ is a random effect for that PK parameter associated with between-individual variability, and κ*_P_*_,*i*,*k*_ is a random effect for the individual parameter associated with within-individual variability.

The residual error for observations was modeled using a combined additional and proportional error model, with ε_prop,*ijk*_ and ε_add,*ijk*_ representing the proportional and additive components of residual variability of tigecycline concentrations. It was assumed that ε_prop_ and ε_add_ variables have normal distribution, with means of 0 and variances of σ^2^_prop_ and σ^2^_add_, respectively.

The covariates considered for testing included the time-independent covariates of age, weight, height, sex, and application of extracorporeal techniques (ECMO and CRRT), as well as the time-dependent covariates of dialysis volume, ultrafiltration (UF) speed, extravascular lung water index (ELWI), cardiac output (CO), sequential organ failure assessment (SOFA) score, and procalcitonin (PCT) concentration. To identify possible relationships, covariate search was performed by plotting random effects of parameters against covariates. For the model including IIV, the estimates of the η values were plotted against time-independent covariates and the median values of time-dependent covariates. For the model including both IIV and IOV, the estimates of the κ values were plotted against time-dependent covariates. As no apparent visual relationship was found, formal statistical testing was not employed.

## Supplementary Material

Supplemental material
