# Supplementary material for: Population Pharmacokinetics of High-Dose Tigecycline in Patients with Sepsis or Septic Shock
Source: Antimicrob Agents Chemother. 2018 Mar 27;62(4):e02273-17. doi: 10.1128/AAC.02273-17 (PMC5913959; doi:10.1128/AAC.02273-17)
Supplement: Supplemental material [file supp_62_4_e02273-17__index.html]

Population Pharmacokinetics of High-Dose Tigecycline in Patients with Sepsis or Septic Shock — Supplemental material 

# Population Pharmacokinetics of High-Dose Tigecycline in Patients with Sepsis or Septic Shock

## Supplemental material

- Supplemental file 1 -

  Supplemental material

  PDF, 2.3M
