# Supplementary material for: Population Pharmacokinetics of High-Dose Tigecycline in Patients with Sepsis or Septic Shock
Source: Antimicrob Agents Chemother. 2018 Mar 27;62(4):e02273-17. doi: 10.1128/AAC.02273-17 (PMC5913959; doi:10.1128/AAC.02273-17)
Supplement: Supplemental material [file AAC.02273-17_zac004187006s1.pdf]

Supplementary material

Chromatographic parameters of the analytical method

The chromatographic separation was carried out using Hypersil BDS C18 (250 x 4.6 mm; 55μm), column (Thermo Scientific, Germany), preceded with a μBondapak C18 Guard-Pak precolumn (Waters, Milford, USA). The mobile phase was composed of 25 : 75 (v/v) acetonitrile – phosphate buffer (pH 3.5, 0.023 M). The ion-pairing agent Triethylamine (10 ml/L) was added to minimize peak tailing and improve peak symmetry. The mobile phase's flow rate was 1.2 mL/min at ambient temperature. The UV detector was set at 225 nm with a sensitivity of 0.01 absorbance units full scale (AUFS). Dionex Chromeleon Chromatography Data System (CDS) software (version 6.30, Scientific software, Sunnyvale, USA) was employed to calculate the peak areas. Peak identification was based on comparison of retention times and diode-array spectra obtained during analytical measurements, with corresponding set of data observed for standard compounds.

Method validation

The linearity of the method for tigecycline's determination was estimated by making three replicate injections of a working standards prepared at six different concentration levels (0.078, 0.156, 0.312, 0.625, 1.25 and 2.5 μg/mL). The internal standard method was used to calculate the calibration curve and tigecycline's plasma concentration. The linear regression equation for comparison of determined and spiked concentration was: $y = 0.8328\ x - 0.2108$, with correlation coefficients of R2 = 0.9984. The precision and accuracy were evaluated by analyzing five replicates of quality control samples at three different concentration levels of tigecycline. Precision was expressed as the coefficient of variation (CVs), though accuracy was presented as a percent error (relative error), [(observed concentration – nominal concentration) / nominal concentration] × 100(%)].

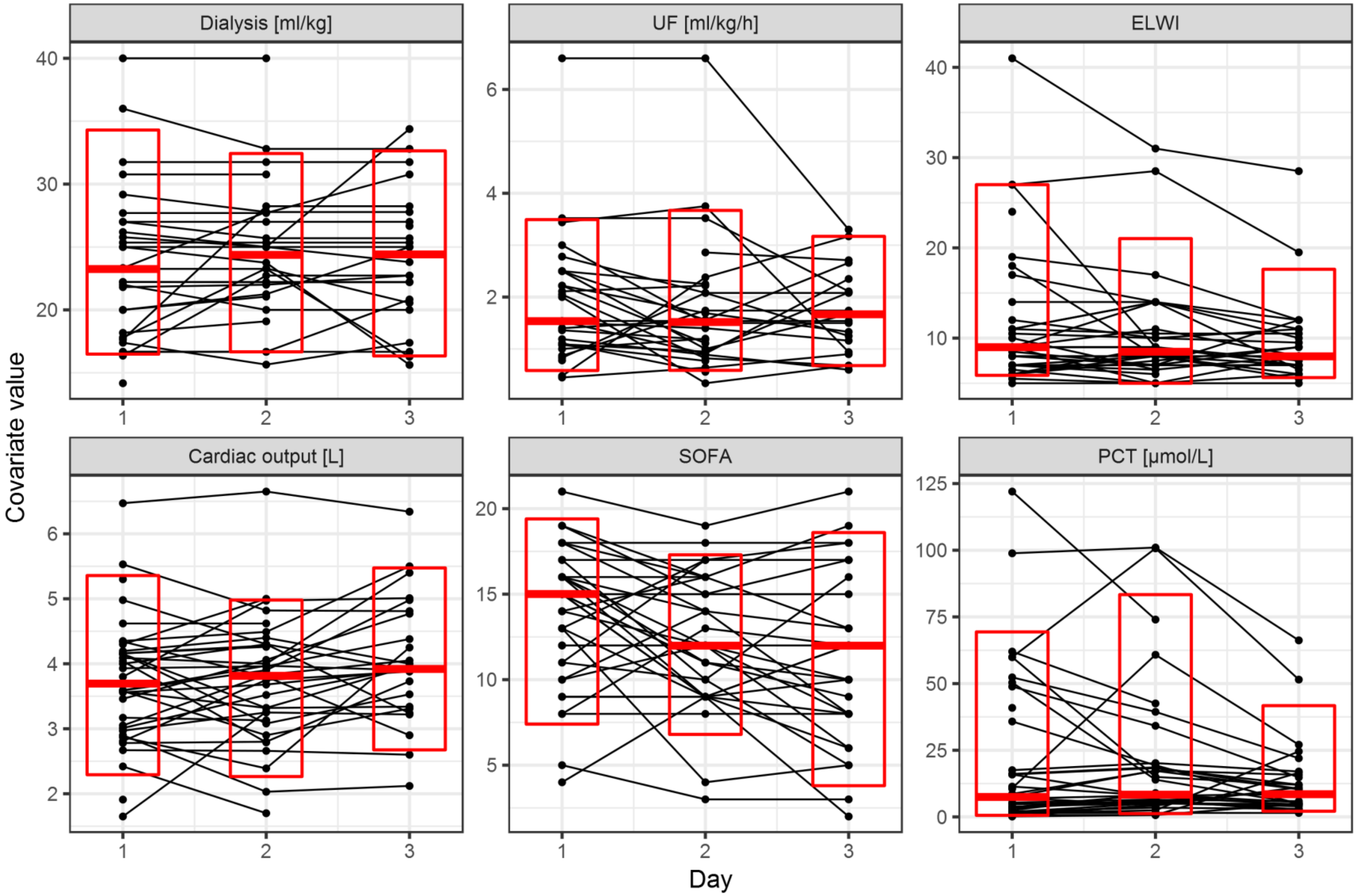

Figure S1. Summary of time-dependent covariates changing over days for the study population. The red boxes cover the 5th-95th percentile range with the red horizontal line denoting the median value.

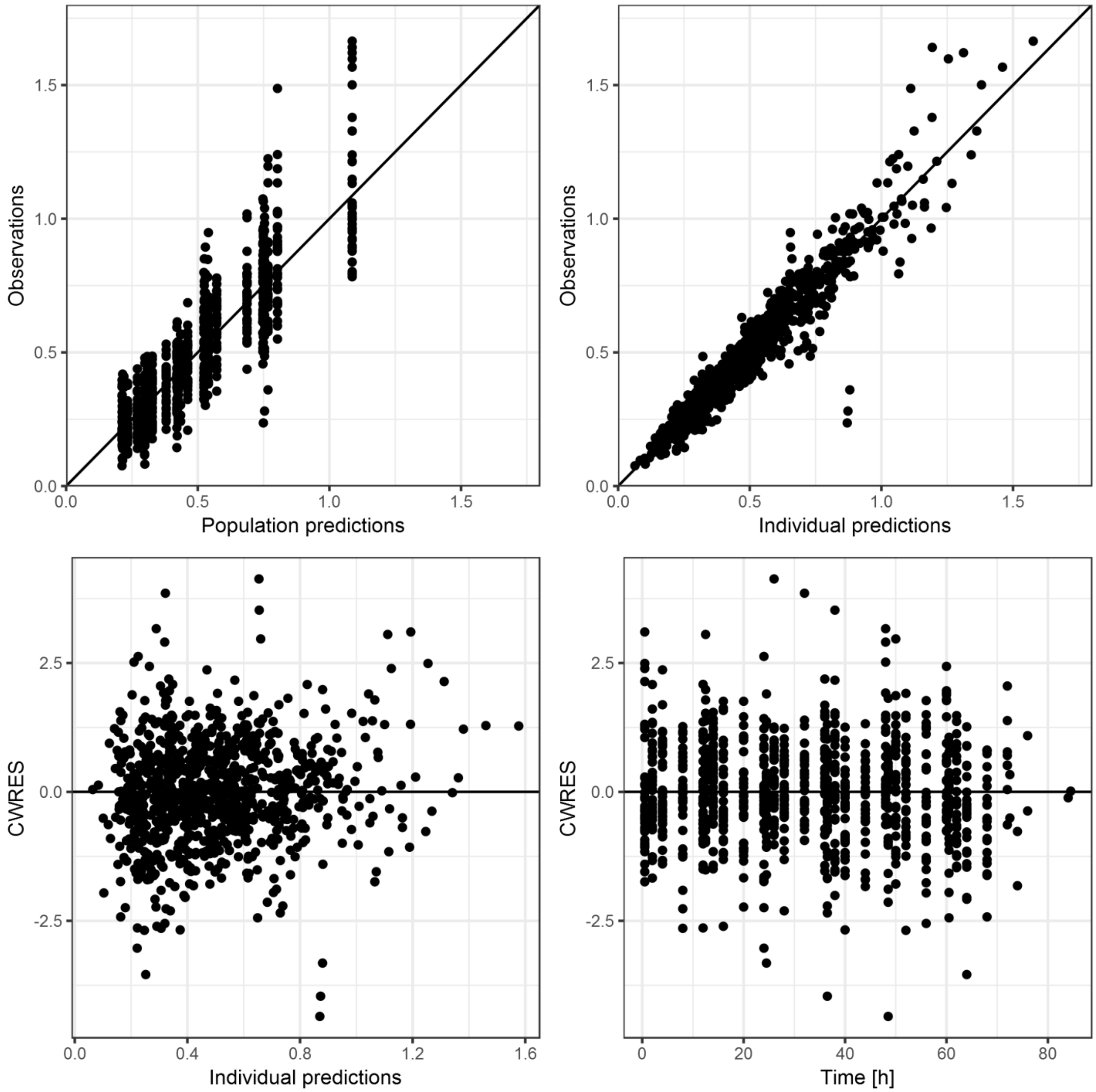

Figure S2. Goodness of fit plots: the observed *versus* the population predicted concentrations; the observed *versus* the individual population predicted concentrations; conditional weighted residuals (CWRES) *versus* individual predicted concentrations and time.

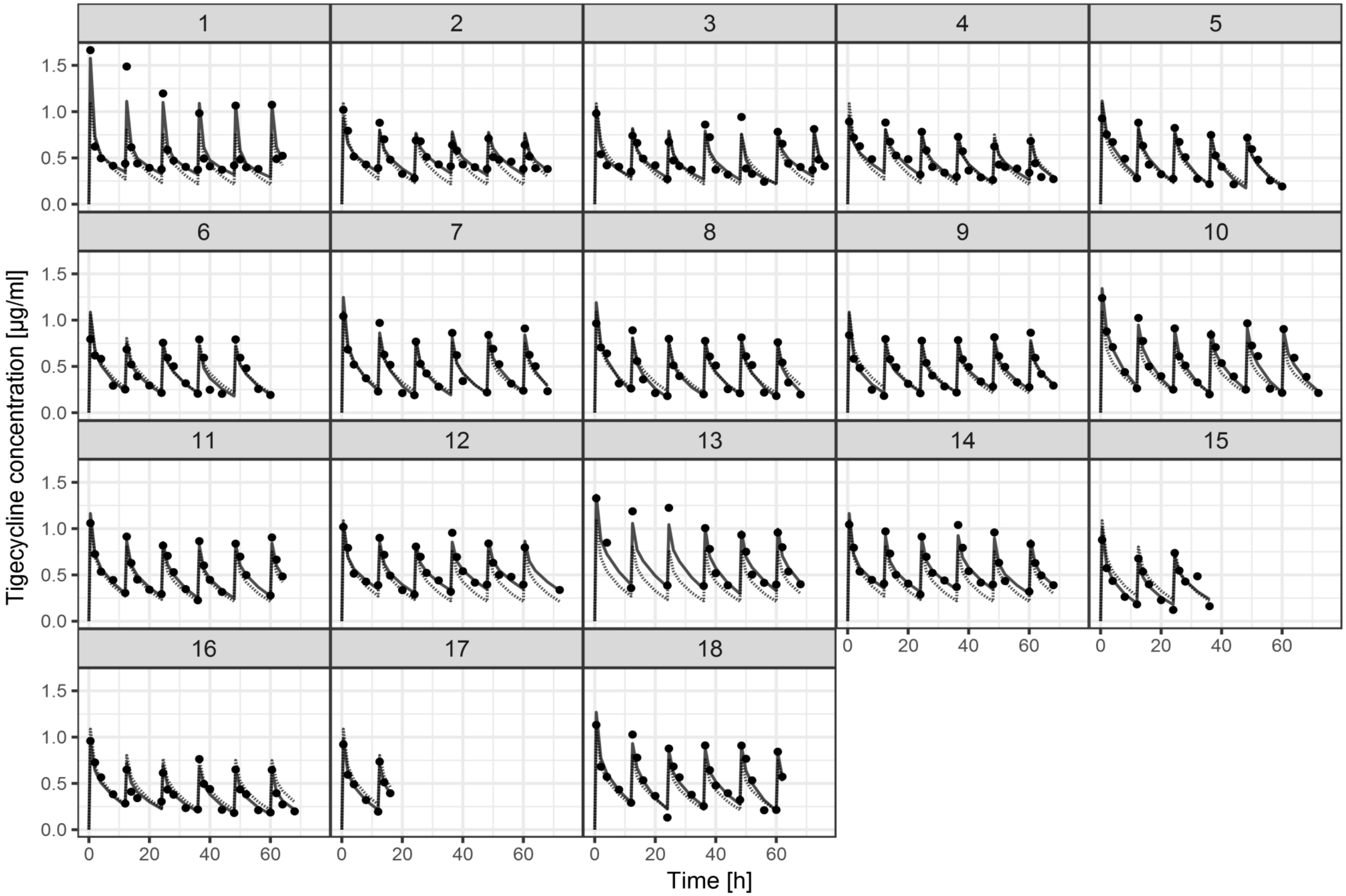

Figure S3a. Experimental (points), individual (solid lines) and population (dashed lines) model predictions of tigecycline concentrations for the patients in the analyzed population.

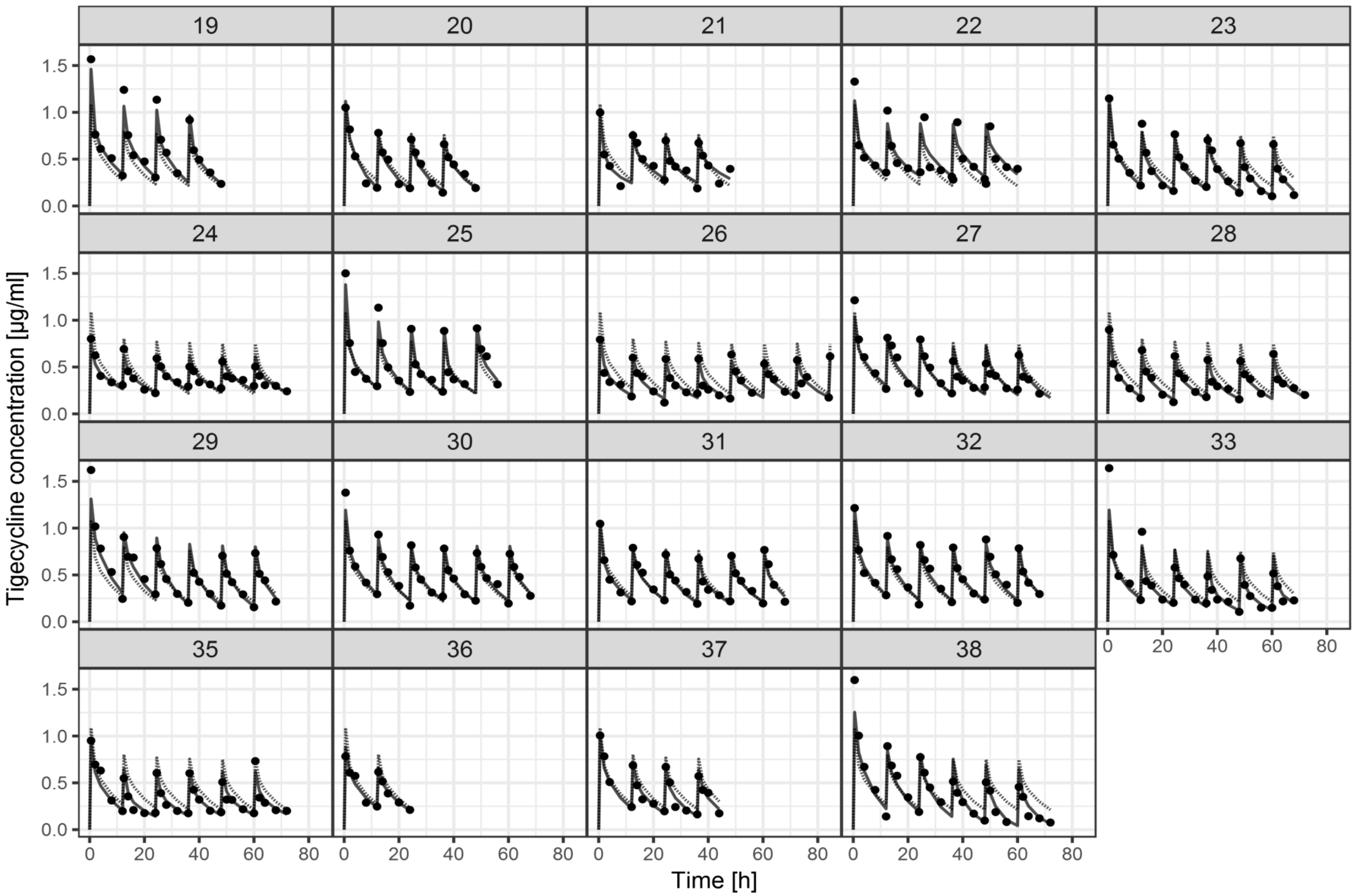

Figure S3b. Experimental (points), individual (solid lines) and population (dashed lines) model predictions of tigecycline concentrations concentrations for the patients in the analyzed population.

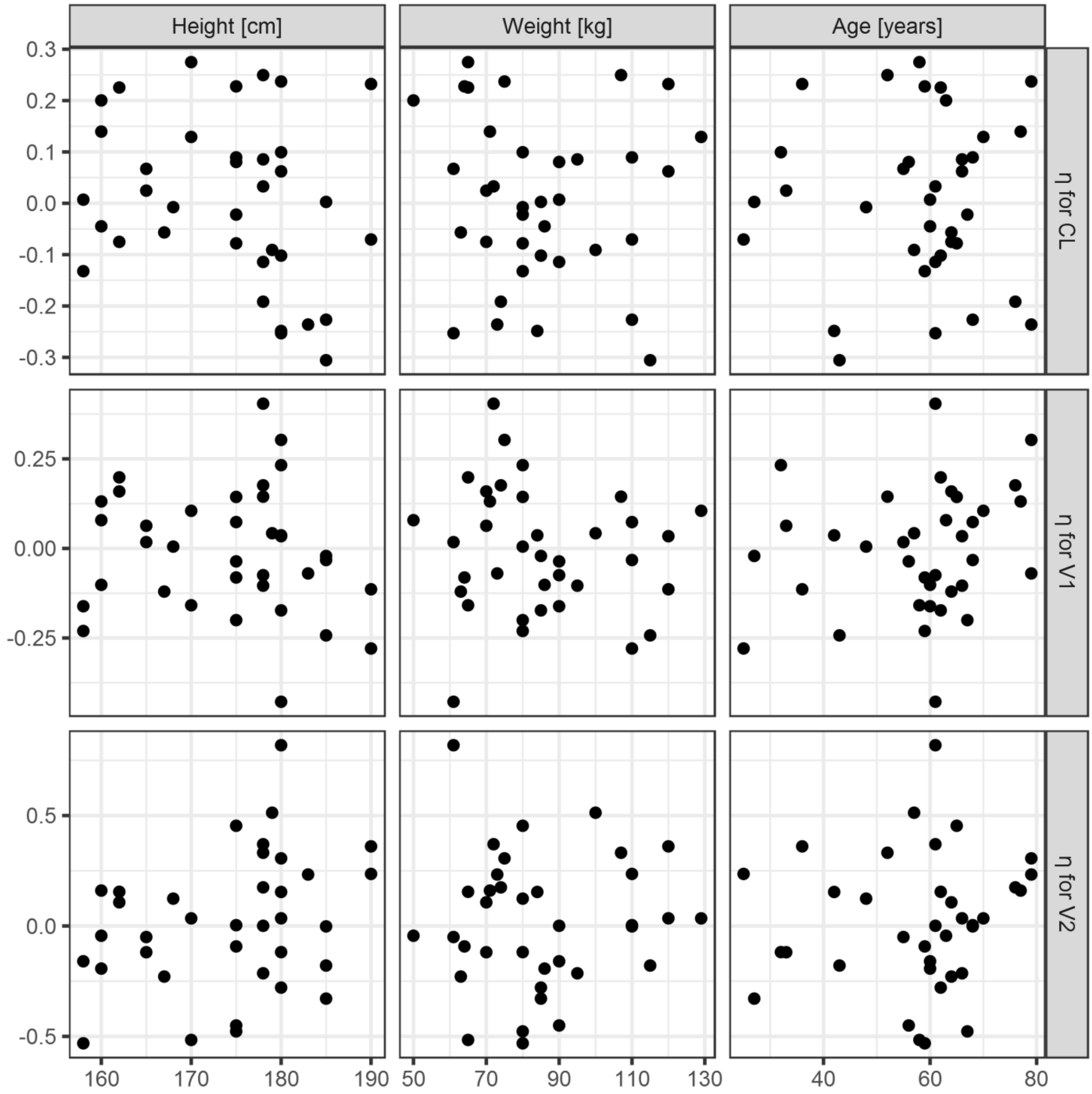

Figure S4. The estimates for eta (deviation of the individual estimate from the population mean) of the final PK parameters in relation to continuous covariates: height, body weight and age.

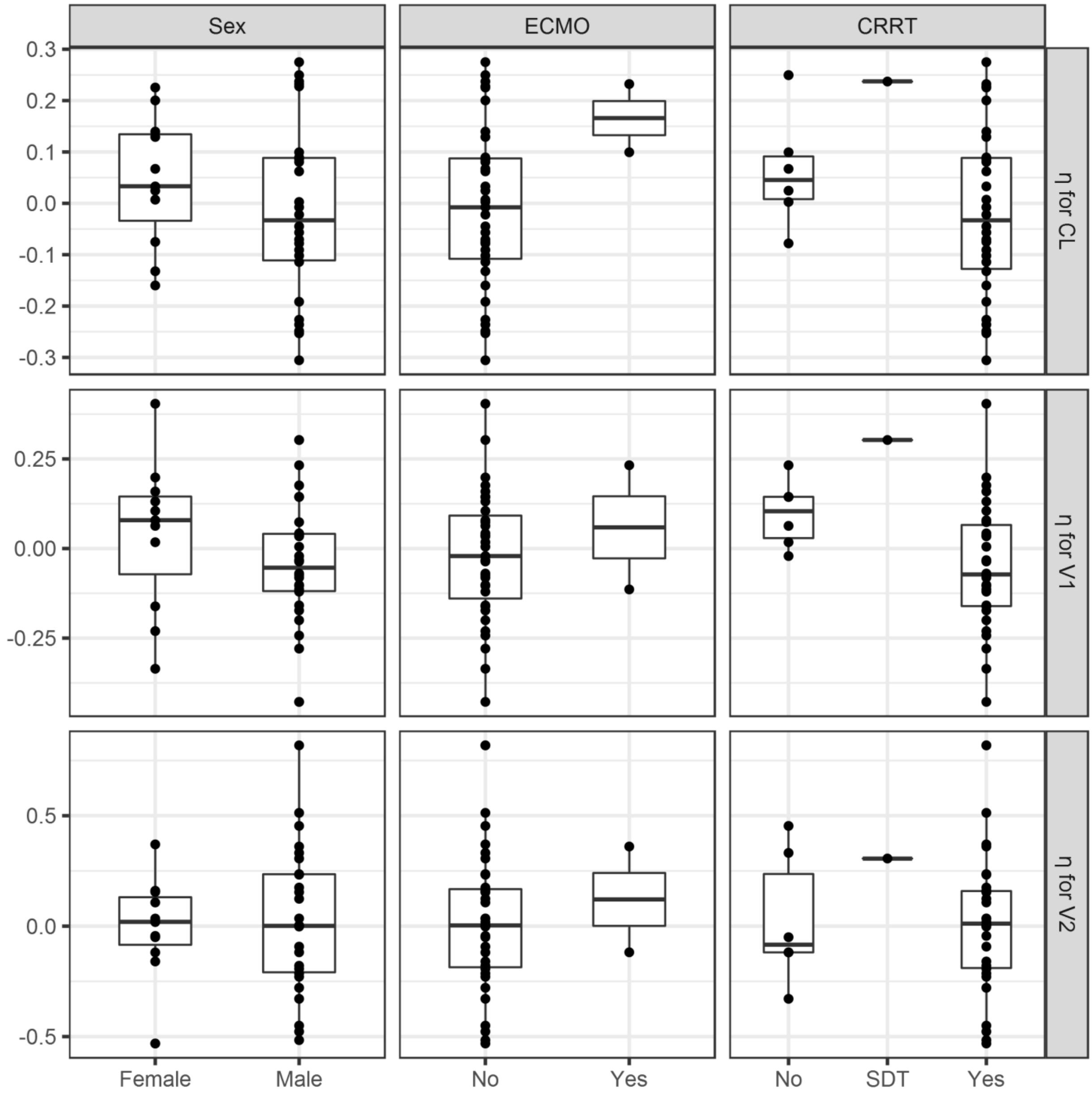

Figure S5. The estimates for eta (deviation of the individual estimate from the population mean) of the final PK parameters in relation to categorical covariates: sex, the use of extracorporeal membrane oxygenation (ECMO) and continuous renal replacement therapy (CRRT; SDT stands for CRRT started during therapy).

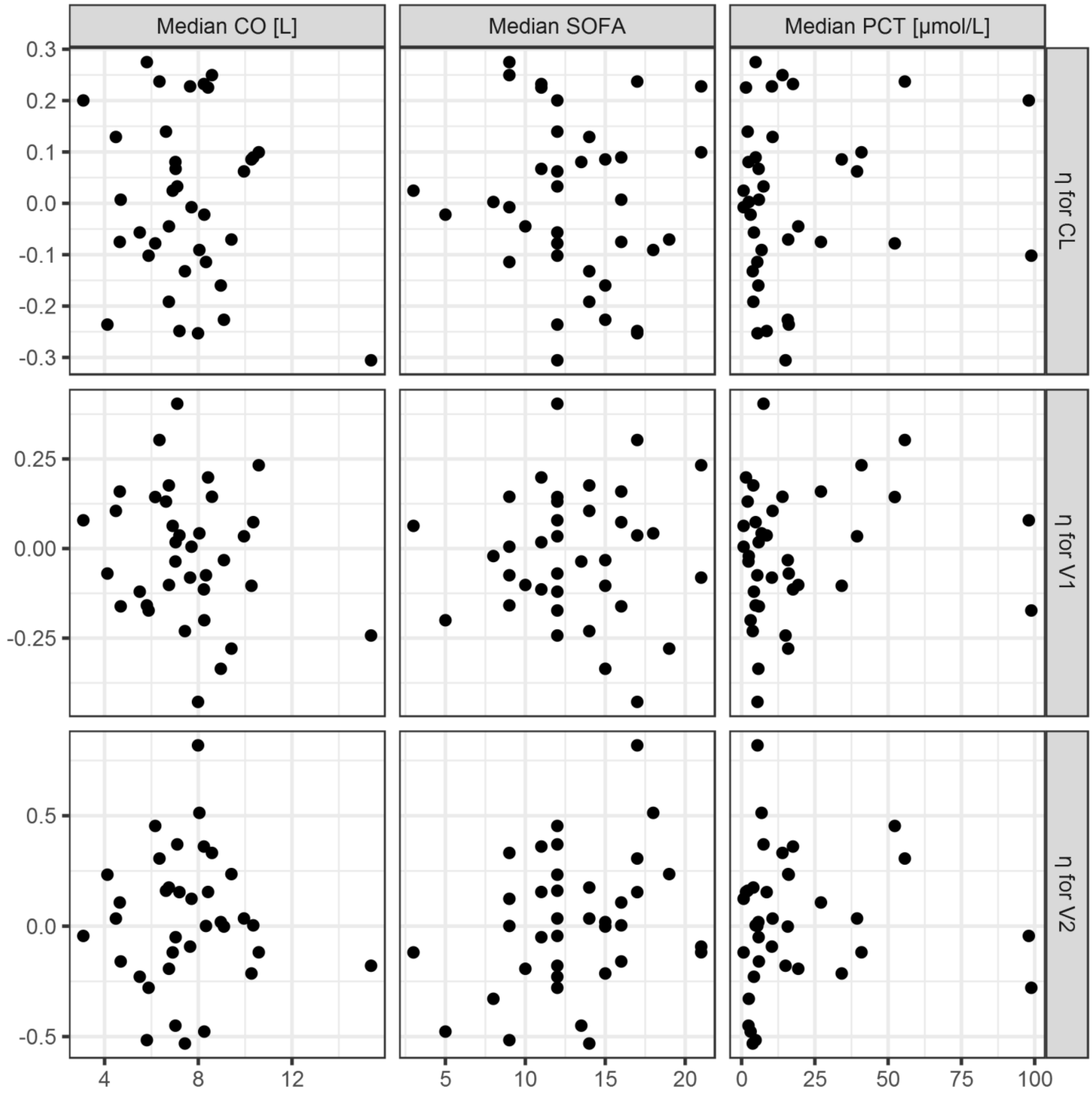

Figure S6a. The estimates for eta (deviation of the individual estimate from the population mean) of the final PK parameters in relation to median values of time-dependent continuous covariates: cardiac output (CO), SOFA score and procalcitonin (PCT) concentration.

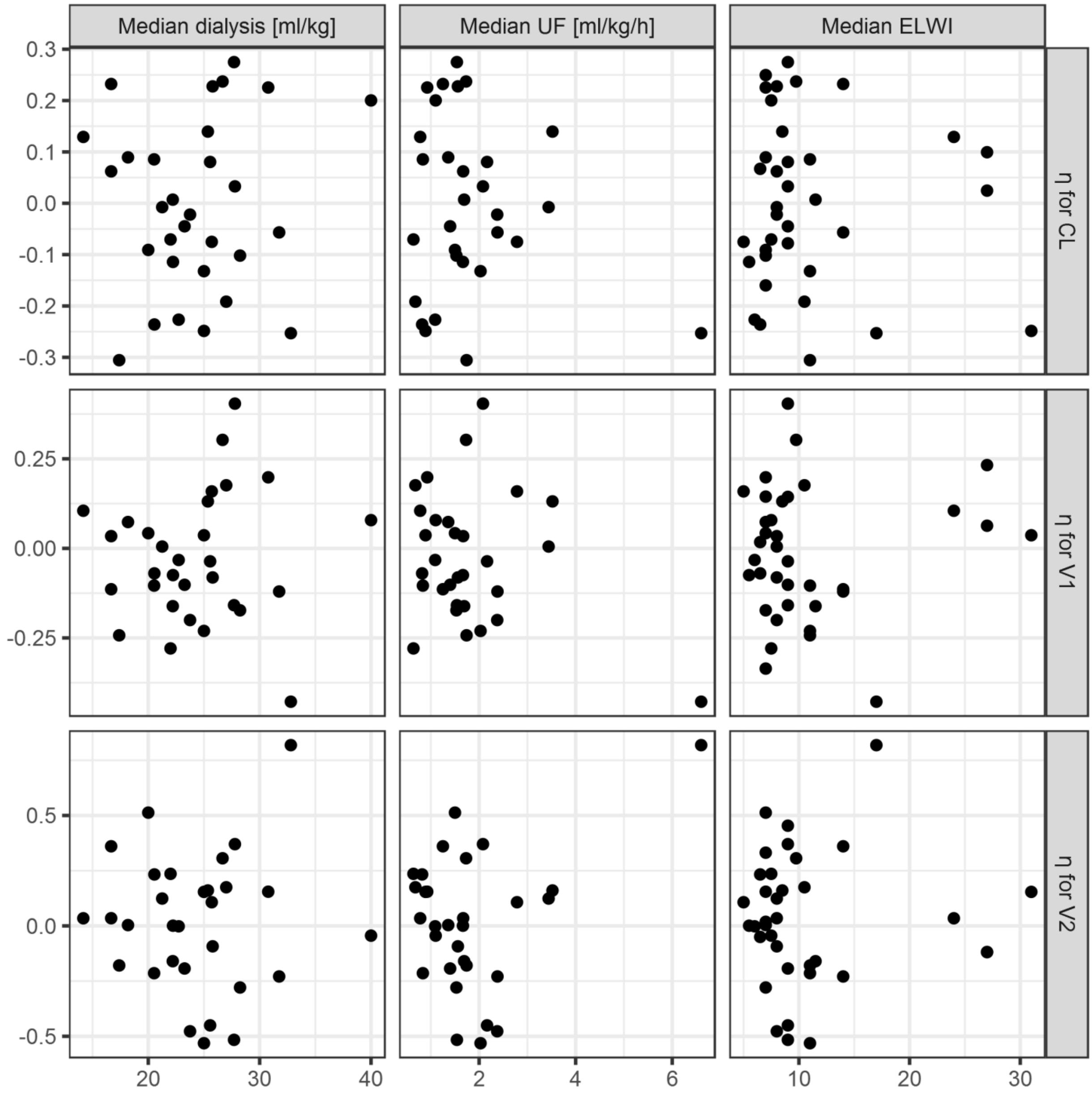

Figure S6b. The estimates for eta (deviation of the individual estimate from the population mean) of the final PK parameters in relation to median values of time-dependent continuous covariates: dialysis volume, ultrafiltration speed, extravascular lung water index (ELWI).
